# Validation of Cell-Cycle Arrest Biomarkers for Acute Kidney Injury after Pediatric Cardiac Surgery

**DOI:** 10.1371/journal.pone.0110865

**Published:** 2014-10-24

**Authors:** Melanie Meersch, Christoph Schmidt, Hugo Van Aken, Jan Rossaint, Dennis Görlich, Dirk Stege, Edward Malec, Katarzyna Januszewska, Alexander Zarbock

**Affiliations:** 1 Department of Anaesthesiology, Intensive Care and Pain Medicine, University of Münster, Münster, Germany; 2 Institute of Biostatistics and Clinical Research, University of Münster, Münster, Germany; 3 Department of Pediatric Cardiology, University of Münster, Münster, Germany; 4 Department of Pediatric Heart Surgery, University of Münster, Münster, Germany; Imperial College London, Chelsea & Westminster Hospital, United Kingdom

## Abstract

**Background:**

The lack of early biomarkers for acute kidney injury (AKI) seriously inhibits the initiation of preventive and therapeutic measures for this syndrome in a timely manner. We tested the hypothesis that insulin-like growth factor-binding protein 7 (IGFBP7) and tissue inhibitor of metalloproteinases-2 (TIMP-2), both inducers of G1 cell cycle arrest, function as early biomarkers for AKI after congenital heart surgery with cardiopulmonary bypass (CPB).

**Methods:**

We prospectively studied 51 children undergoing cardiac surgery with CPB. Serial urine samples were analyzed for [TIMP-2]•[IGFBP7]. The primary outcome measure was AKI defined by the pRIFLE criteria within 72 hours after surgery.

**Results:**

12 children (24%) developed AKI within 1.67 (SE 0.3) days after surgery. Children who developed AKI after cardiac surgery had a significant higher urinary [TIMP-2]•[IGFBP7] as early as 4 h after the procedure, compared to children who did not develop AKI (mean of 1.93 ((ng/ml)^2^/1000) (SE 0.4) vs 0.47 ((ng/ml)^2^/1000) (SE 0.1), respectively; p<0.05). Urinary [TIMP-2]•[IGFBP7] 4 hours following surgery demonstrated an area under the receiver-operating characteristic curve of 0.85. Sensitivity was 0.83, and specificity was 0.77 for a cutoff value of 0.70 ((ng/ml)^2^/1000).

**Conclusions:**

Urinary [TIMP-2]•[IGFBP7] represent sensitive, specific, and highly predictive early biomarkers for AKI after surgery for congenital heart disease.

**Trial Registration:**

www.germanctr.de/, DRKS00005062

## Introduction

Acute kidney injury (AKI) is a common complication of pediatric cardiac surgery and negatively impacts short- and long-term outcomes [1]–[3]. Serum creatinine (S_Cr_), the traditional marker of renal function, does not rise appreciably before a 50% loss in glomerular filtration rate (GFR) had occurred. Furthermore, S_Cr_ is affected by several non-renal factors. Due to hemodilution during CPB, S_Cr_ levels do not peak until 1 to 3 days after cardiac surgery [4]. The other traditional marker of renal function, urine output, has a low specificity after cardiac surgery, because it is also influenced by several factors. Thus, our ability to detect AKI early remains insufficient.

The failure of prior interventional trials to attenuate AKI after cardiac surgery has been attributed in part to delays in the diagnosis of AKI [5], [6]. Currently, it is believed that progress in this field is forthcoming with the availability of new biomarkers for early and reliable prediction of AKI [7], [8]. Initial human studies demonstrate that urine tissue inhibitor of metalloproteinases-2 (TIMP-2) and insulin-like growth factor-binding protein 7 (IGFBP7), both inducers of G1 cell cycle arrest, are early markers of AKI in critically ill patients [9], [10]. In a recent adult study, we also showed that TIMP-2 and IGFBP7 are elevated 24 to 48 hours before the clinical diagnosis of cardiac surgery-associated AKI becomes apparent [11].

In this preliminary study, we have tested the hypothesis that urinary [TIMP-2]•[IGFBP7] can predict AKI in pediatric patients undergoing congenital heart surgery earlier than currently used clinical parameters. The primary end point of our study was the development of AKI as defined by pediatric modified RIFLE (pRIFLE) criteria [12].

## Materials and Methods

### Study Design

This study was approved by the Institutional Review Board of University of Münster. We used the Standards for Reporting of Diagnostic Accuracy (STARD) statement for planning and conducting the study and preparing the manuscript [13].

All patients <18 years of age undergoing cardiac surgery with cardiopulmonary bypass (CPB) at our center between July 2013 and December 2013 were approached for study inclusion. Patients with severe pre-existing renal insufficiency (S_Cr_ >2 times age-adjusted normal range) were excluded. Written informed consent was obtained before enrollment from the legal guardian of each patient with assent from the patient when appropriate.

Urine samples for biomarker analysis were obtained immediately before and at 4, and 24 hours after initiation of CPB, and stored in aliquots at −80°C. S_Cr_ was routinely measured before surgery, immediately after surgery and at least daily in the post-operative period.

The primary outcome was the development and severity of AKI as defined by the pediatric modified RIFLE (pRIFLE) criteria within 72 hours after cardiac surgery [12]. We determined pRIFLE by calculation of estimated creatinine clearance (eCCl) using the modified Schwartz formula [14], with “Risk” defined as eCCL decrease of 25% from baseline, “Injury” defined as eCCl decrease of 50%, and “Failure” defined as eCCl decrease of 75% or absolute value <35 ml/min/1.73 m^2^. Complexity of surgery was categorized according to the Risk Adjustment for Congenital Heart Surgery 1 (RACHS-1) consensus-based scoring system [15]. Secondary outcomes included duration of mechanical ventilation, hospital length of stay and hospital mortality.

### Biomarker Measurements

Laboratory investigators were blinded to clinical outcomes. Urine TIMP-2 and IGFBP7 were measured with the NephroCheck Test (Astute Medical, San Diego, CA, USA). The NephroCheck Test is a point-of-care test which was developed to simultaneously measure urine [TIMP-2]•[IGFBP7], whereas [TIMP-2]•[IGFBB7] indicates the multiplication of both biomarkers. Urine neutrophil gelatinase-associated lipocalin (NGAL) was assayed using a human-specific commercially available ELISA (AntibodyShop, Grusbakken, Denmark). The urine kidney injury molecule (KIM)-1 ELISA was constructed using commercially available reagents (R&D Systems, Inc., Minneapolis, MN).

### Statistical Methods

**Figure 1:**
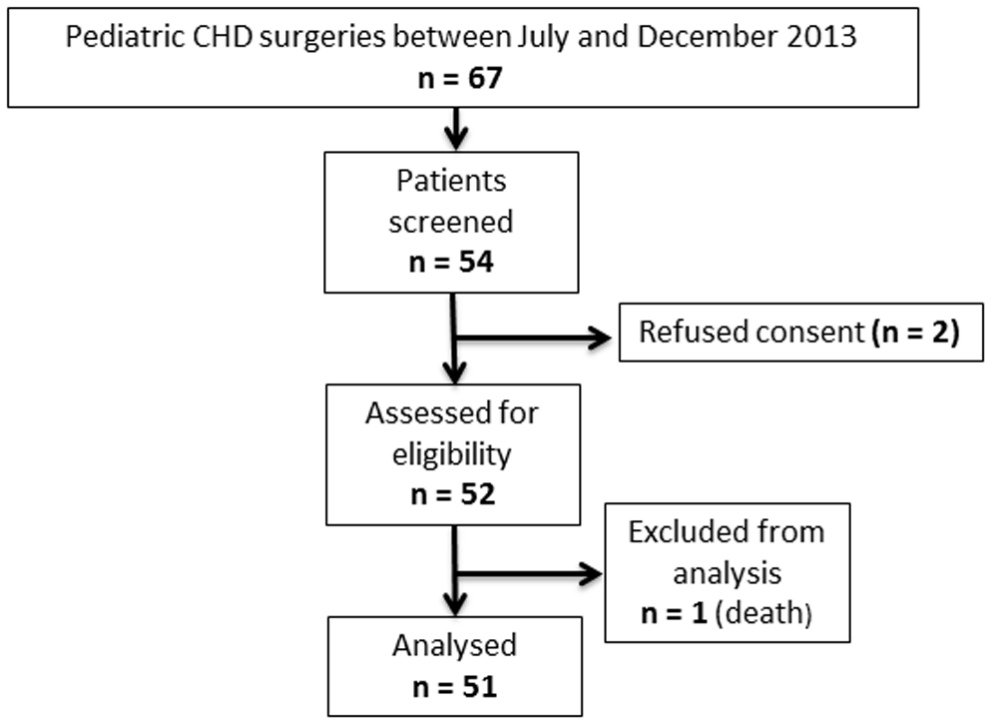
Flow Diagram.

For the primary analysis, that is the difference between the urine [TIMP-2]*[IGFBP7] levels in patients with AKI or without AKI, we applied a Mann-Whitney-U-test. Based on the published results on [TIMP-2]*[IGFBP7] [9], we aimed to detect a difference in 1 unit in [TIMP-2]*[IGFBP7] with a power of 90%. Assuming an effect size of 1, a sample size of 52 patients is necessary. Power calculation was performed with nQuery Advisor (Version 7). Thus, 52 patients were prospectively included in the protocol (Figure 1).

The analysis subset included patients who had measurement of all three biomarkers at the different time points to permit comparisons of biomarkers. Statistical analysis was performed using SPSS (IBM SPSS Statistics for Windows, Version 21.0. Armonk, NY: IBM Corp.). Demographics, baseline measurements and clinical outcomes were compared between AKI and non-AKI patients using the Mann-Whitney-U test (continuous variables) or χ^2^ or Fisher's exact tests (categorical variables) as appropriate.

To analyze the predictive power of selected biomarkers receiver operating characteristic curves (ROC) were calculated and the area under the ROC curve (AUC) was determined. 95% confidence intervals (CI) were reported. The AUCs were compared between biomarkers using the methods developed by DeLong [16]. For selected thresholds of [TIMP2]•[IGFBP7] sensitivities, specificities, positive predictive values (PPV) and negative predictive values (NPV) were reported for each time point.

To include the effect of [TIMP2]•[IGFBP7] into a time-to-AKI model we used a cox-proportional hazard model. We model the time until an AKI occurs in terms of hours and assume all non-AKI patients to be censored for the analysis. For censored patients time until censoring is calculated as time from end of surgery cardiac surgery until end of observation (three days). At first, a clinical model without biomarkers was estimated from the data using relevant clinical factors. In a second step, the [TIMP2]•[IGFBP7] level was included. To show the benefit of the extended model (clinical model + [TIMP2]•[IGFBP7]) we report the result of the likelihood ratio test between the two models.

Inferential statistics are intended to be exploratory (hypotheses generating), not confirmatory, and are interpreted accordingly. The comparison-wise type-I error rate is controlled instead of the experiment-wise error rate. The local significance level is set to 0.05. No adjustment for multiple testing is performed.

## Results

We enrolled 51 patients (Figure 1). There was no significant difference regarding the age and baseline S_Cr_ (Table 1) between patients who developed an AKI and who did not develop an AKI. CPB time, duration of mechanical ventilation and hospital LOS were similar between AKI and non-AKI groups. Baseline urinary biomarker concentrations were not different in AKI and non-AKI groups (Table 1).

**Table 1:** Patients Characteristics.

Characteristics	No AKI (n = 39)	AKI (n = 12)	p value
Age, yrs	3±0.5	1.5±1.0	0.435
Male	29 (57)	8 (16)	0.715
Prior surgery	33 (65)	8 (16)	0.218
Bypass time, min	78±9	107±15	0.130
Baseline SCr, mg/dl	0.5±0.1	0.4±0.1	0.107
Baseline eCCL, ml/min/1.73 dm^2^	123±7	141±14	0.232
Baseline urine [TIMP-2]•[IGFBP7], ((ng/ml)^2^/1000)	1.0±0.1	0.9±0.3	0.818
Baseline urine NGAL, ng/ml	10±5	10±4	0.949
Baseline urine KIM-1, pg/ml	146±67	145±68	0.991
Duration of preoperative fasting, hours	5.6±0.1	5.5±0.3	0.599
Hospital stay, days	15±1	20±3	0.119
ICU stay, day	3±0.4	4±1.7	0.332
Ventilator, hours	9.5±6	12±9	0.332
Death	0 (0)	0 (0)	0.584
RACHS1 score			0.391
1	2	0	
2	12	6	
3	25	6	
pRIFLE			
R	0	10	
I	0	2	
F	0	0	
Dialysis	0 (0)	0 (0)	0.584

Mean ±SE reported for continuous variables, p-values from Mann-Whitney-U test. Frequency (proportion) reported for categorical variables, with p-values from χ^2^ test or Fisher's exact test. pRIFLE reported for AKI patients only.

**Figure 2:**
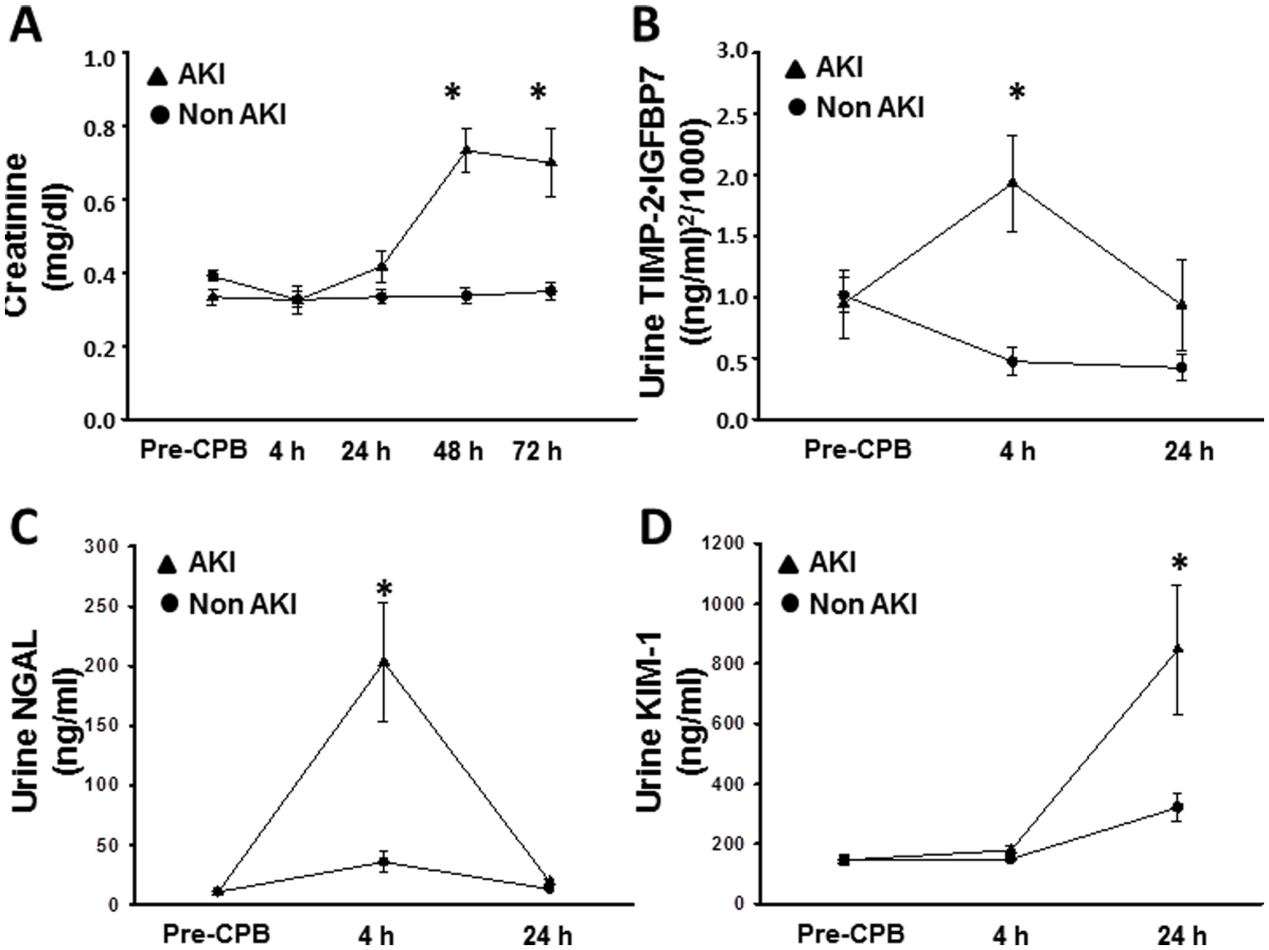
Analysis of urine biomarkers. (A) Graph shows creatinine concentrations in the plasma at various time points before and after cardiopulmonary bypass. (B and C) Graph shows mean urine [TIMP-2]*[IGFBP7] (B) and neutrophil gelatinase-associated lipocalin (NGAL) (C) concentrations at various time points before and after cardiopulmonary bypass. Error bars are SE. Asterisks (*) denote significant differences (p≤0.05, Mann-Whitney-U test) between groups (AKI, non-AKI) at the respective time point. (D) Graph shows mean urine kidney injury molecule (KIM)-1 concentrations at various time points before and after cardiopulmonary bypass. Error bars are SE. Asterisks (*) denote significant differences (p≤0.05, Mann-Whitney-U test) between groups (AKI, non-AKI) at the respective time point.

AKI occurred in 12 (24%) of the 51 patients 1.67 (SE 0.3) days after surgery. Patient characteristics are shown in Table 1. In patients who developed AKI, S_Cr_ significantly increased from 0.3 mg/dl (SD 0.1) at baseline to 0.7 mg/dl (SD 0.2) two days after surgery, whereas S_Cr_ in patients who did not develop AKI remained unchanged (Figure 2A). Among AKI patients, 10 patients developed pRIFLE-R, 2 patients developed pRIFLE-I, and none of the patients developed pRIFLE-F.

In the 39 patients who never developed AKI, a significant decrease in urinary [TIMP-2]•[IGFBP7] level after CPB was noted as compared to the preoperative measurement (p<0.01; Figure 2B). By contrast, those who subsequently developed AKI had a striking rise in urinary [TIMP-2]•[IGFBP7] level 4 h after the procedure as compared to the pre-CPB values (Figure 2B). The pattern of urinary [TIMP-2]•[IGFBP7] excretion was characterized by a peak very early after the precipitating event followed by a strong decrease (Figure 2B). The neutrophil gelatinase-associated lipocalin (NGAL) concentration in the urine of patients who developed an AKI significantly increased 4 hours after surgery followed by a sharp decrease (Figure 2C), whereas kidney injury molecule (KIM)-1 in the urine significantly increased at a later time point (Figure 2D).

In our study population, baseline [TIMP-2]•[IGFBP7] levels were not elevated in patients with either immature kidneys (<2 years) or venous congestion resulting from congenital heart disease (data not shown), suggesting that [TIMP-2]•[IGFBP7] serves as a marker of acute injury to the kidney after cardiac surgery with CPB.

**Figure 3:**
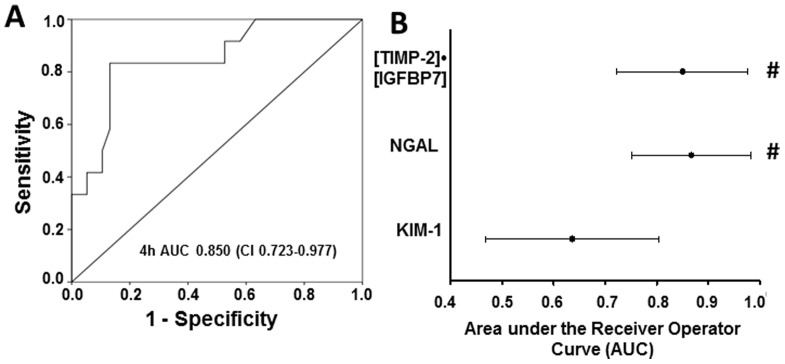
Analysis of ROC curves. (A) This figure displays the receiver operating characteristic (ROC) curves for the 4 h value of [TIMP-2]•[IGFBP7]. (B) Area under the receiver-operating characteristics curve (AUC) for [TIMP-2]•[IGFBP7] and existing biomarkers of acute kidney injury. The AUC for urinary [TIMP-2]•[IGFBP7] is as large as the AUC for urinary NGAL and significantly larger than the AUC for urinary kidney injury marker-1 (KIM-1).^#^ p<0.05 vs KIM-1.

For urine [TIMP-2]•[IGFBP7], the area under the ROC curve was 0.85 (CI: 0.72–0.94) at 4 h after CPB (Figure 3A), the area under the ROC curve for urine NGAL was 0.87 (CI: 0.74–0.95) (Figure 3B), and the area under the ROC curve for urine KIM-1 was 0.64 (CI: 0.49–0.77) (Figure 3B). Furthermore, the AUCs of biomarkers were compared. There was no significant difference between the AUC of NGAL and [TIMP2]•[IGFBP7] (p = 0.8549). The AUCs of [TIMP2]•[IGFBP7] and NGAL were significant higher compared to the AUC of KIM-1 ([TIMP2]•[IGFBP7] vs KIM-1: p = 0.0326, NGAL vs KIM-1: p = 0.0280) (Figure 3B). Table 2 lists the derived sensitivities, specificities, and predictive values at different cutoff concentrations. For urine [TIMP-2]•[IGFBP7], a cutoff of 0.7 yielded good sensitivity and specificity at 4 h after CPB.

**Table 2:** [TIMP2]•[IGFBP7] test characteristics at different cutoff values.

	Sensitivity	Specificity	PPV	NPV
**4 h**				
0.3 ((ng/ml)^2^/1000)	0.83	0.64	0.42	0.93
0.4 ((ng/ml)^2^/1000)	0.83	0.67	0.43	0.93
0.5 ((ng/ml)^2^/1000)	0.83	0.69	0.45	0.93
0.6 ((ng/ml)^2^/1000)	0.83	0.74	0.50	0.94
0.7 ((ng/ml)^2^/1000)	0.83	0.77	0.52	0.94
**24 h**				
0.3 ((ng/ml)^2^/1000)	0.50	0.54	0.25	0.78
0.4 ((ng/ml)^2^/1000)	0.50	0.67	0.32	0.81
0.5 ((ng/ml)^2^/1000)	0.50	0.77	0.40	0.83
0.6 ((ng/ml)^2^/1000)	0.50	0.79	0.43	0.84
0.7 ((ng/ml)^2^/1000)	0.50	0.79	0.43	0.84

PPV, positive predictive value; NPV, negative predictive value.

We also tested whether [TIMP-2]•[IGFBP7] increases predictive ability over common clinical variables. [TIMP-2]•[IGFBP7] significantly strengthened risk prediction when added to a seven-parameter clinical model for our primary endpoint using time-to-event analysis (Table 3). This analysis showed significant enhancement by the addition of [TIMP-2]•[IGFBP7] with [TIMP-2]•[IGFBP7] remaining strongly associated with AKI in this model.

**Table 3:** Cox Proportional Hazards Models for [TIMP-2]•[IGFBP7] (4 h) and clinical covariates.

	Clinical Model		Clinical Model with [TIMP-2]•[IGFBP7]^3^	
Variable^1^	Hazard Ratio^2^	p-value	Hazard Ratio^2^	p-value
Gender	1.414 (0.320–6.246)	0.648	4.666 (0.647–33.627)	0.126
Age	1.000 (0.998–1.003)	0.683	0.998 (0.994–1.002)	0.380
Ventilation time	0.997 (0.979–1.015)	0.739	0.987 (0.946–1.030)	0.557
Bypass time	1.007 (0.998–1.016)	0.121	1.005 (0.996–1.014)	0.320
Prior surgery	0.535 (0.139–2.057)	0.363	0.467 (0.066–3.279)	0.444
BMI	1.101 (0.835–1.451)	0.496	1.307 (0.904–1.889)	0.154
RACHS-1 score	0.722 (0.211–2.477)	0.605	1.069 (0.257–4.446)	0.927
TIMP-2*IGFBP7 (4 h)	Not included in model		2.872 (1.569–5.258)	0.001

1 reference category (underlined) for gender is male vs female, for RACHS score is 1+2 vs 3 and prior surgery no vs. yes. Age, ventilation time, BMI and [TIMP-2]•[IGFBP7] are included as continuous variables. Wald test. P values are reported.

2 95%-Confidence interval given in brackets;

3 Adding [TIMP-2]•[IFGBP7] improves the model significantly (p = 0.0002, likelihood ratio test).

## Discussion

To our knowledge, this is the first study investigating [TIMP-2]•[IGFBP7] as early predictive biomarker of cardiac-surgery associated AKI in children with congenital heart disease (CHD). In our preliminary study, we show that [TIMP-2]•[IGFBP7] levels and NGAL levels increase as early as 4 h after CPB in children who will later develop AKI but not in those who will not develop AKI. AUCs above 0.85 indicate a better performance than other new biomarkers.

Several molecules are involved in the pathogenesis of AKI [17], [18]. IGFBP7 and TIMP-2 are two molecules which may induce G_1_ cell cycle arrest, a mechanism involved in the early phase of AKI [19], [20]. After stress, cell damage or injury, renal tubular cells enter for a short period G_1_ cell-cycle arrest [21] until the damage has been repaired [22]. Importantly, TIMP-2 and IGFBP7 are able to signal in an autocrine as well as paracrine fashion [23]–[27] and thus spread the ‘alarm-signal’ from the site of cell injury. Cell cycle arrest happens early after a variety of insults [20]. This may explain the early increase of these two molecules in the urine of patients who develop AKI after cardiac surgery.

Compared to adult patients at high risk for AKI undergoing cardiac surgery [11], pediatric patients display higher baseline urinary [TIMP-2]•[IGFBP7] concentrations, an observation that can hardly be explained by either immaturity of childrens' kidneys or venous congestion in consequence of CHD. Apart from immaturity and venous congestion preoperative fasting may play a decisive role, as it provokes preoperative dehydration with reduced kidney perfusion. Although we did not find a significant difference in perioperative fasting between the two groups, this hypothesis has to be tested in further larger studies. Other biomarkers which have been scrutinized in the setting of CHD (e.g. KIM-1 and NGAL) do not change in non-AKI children [28]. However, contrary to expectations [TIMP-2]•[IGFBP7] levels decrease after surgery in these children. This decline may suggest that CHD is uniformly associated with a certain degree of kidney injury. Against this background, to test urinary [TIMP-2]•[IGFBP7] seems to provide the best means to diagnose and follow kidney dysfunction in the wake of CHD and surgery.

Our study has limitations. In a single center study, we merely looked at a small number of patients out of the broad spectrum of CHD. Although some parameters (e.g. CPB time, duration of mechanical ventilation and hospital length of stay) were not statistically significant between the groups, it is possible that there are differences, but we could not detect them because of the small sample size. The same may be true for the missing difference in the baseline [TIMP-2]•[IGFBP7] levels in patients with either immature kidneys (<2 years) or venous congestion resulting from congenital heart disease. For the clinical model we selected factors that are normally associated with the occurrence of AKI. However, in this study these factors were not associated with the occurrence of AKI. The reason for this might be the small sample size. The results of this pilot study have to be confirmed in a larger study. Further thorough study is needed to more clearly delineate the interactions between kidney dysfunction and specific congenital cardiac lesions on the one hand and the respective performance of biomarkers on the other hand.

In summary, our preliminary study indicates that [TIMP-2]•[IGFBP7] has the potential to become a highly useful biomarker to early predict AKI in children undergoing cardiac surgery.
